# Crystallization of Esomeprazole Magnesium Water/Butanol Solvate

**DOI:** 10.3390/molecules21040544

**Published:** 2016-04-23

**Authors:** Jenna Skieneh, Bahareh Khalili Najafabadi, Stephen Horne, Sohrab Rohani

**Affiliations:** 1 Department of Chemical and Biochemical Engineering, The University of Western Ontario, London, ON N6A 3K7, Canada; jskieneh@uwo.ca (J.S.); bkhalili@uwo.ca (B.K.N.); shornehome@gmail.com (S.H.); 2 Apotex Pharmachem Inc., 34 Spalding Drive, Brantford, ON N3T 6B8, Canada

**Keywords:** pharmaceutical solvates, solid-state analysis, single crystal X-ray diffraction, thermal analysis, crystallization

## Abstract

The molecular structure of esomeprazole magnesium derivative in the solid-state is reported for the first time, along with a simplified crystallization pathway. The structure was determined using the single crystal X-ray diffraction technique to reveal the bonding relationships between esomeprazole heteroatoms and magnesium. The esomeprazole crystallization process was carried out in 1-butanol and water was utilized as anti-solvent. The product proved to be esomeprazole magnesium tetrahydrate with two 1-butanol molecules that crystallized in P6_3_ space group, in a hexagonal unit cell. Complete characterization of a sample after drying was conducted by the use of powder X-ray diffraction (PXRD), ^1^H-nuclear magnetic resonance (NMR), thermogravimetric analysis (TGA), differential scanning calorimetry (DSC), infrared spectroscopy (IR), and dynamic vapor sorption (DVS). Investigation by ^1^H-NMR and TGA has shown that the solvent content in the dried sample consists of two water molecules and 0.3 butanol molecules per esomeprazole magnesium molecule. This is different from the single crystal X-ray diffraction results and can be attributed to the loss of some water and 1-butanol molecules stabilized by intermolecular interactions. The title compound, after drying, is a true solvate in terms of water; conversely, 1-butanol fills the voids of the crystal lattice in non-stoichiometric amounts.

## 1. Introduction

Esomeprazole, the (*S*)-enantiomer of omeprazole, is the first single optical isomer proton pump inhibitor [1]. This eutomer forms an active inhibitor achiral sulphenamine when in the acidic compartment of the parietal cell. The activated form of omeprazole prevents the formation of gastric acid by blocking the enzyme H^+^/K^+^-ATPase, which is accountable for the formulation of gastric acid and is found in the secretory membranes of the parietal cell [2]. The stability of this active pharmaceutical ingredient (API) is a function of pH and acidic conditions must be avoided. In acidic conditions, omeprazole can degrade to form at least five distinct impurities. These degradants do not inhibit the production of gastric acid and reduce the competence of the pharmaceutical [3]. Omeprazole has been documented to decompose up to 10% in air due to moisture [4]. Esomeprazole has been presented in various forms of alkaline salts as a means of increasing stability. The salt of interest is esomeprazole magnesium, which has a half-life of 19 h at 25 °C and 8 h at 37 °C while at pH 6 [5]. In literature, there are existing examples containing the single crystal structures for omeprazole and toluene hemisolvate piperazinium esomeprazolate; however, the molecular structure of esomeprazole magnesium is unknown [6,7,8].

**Figure 1:**
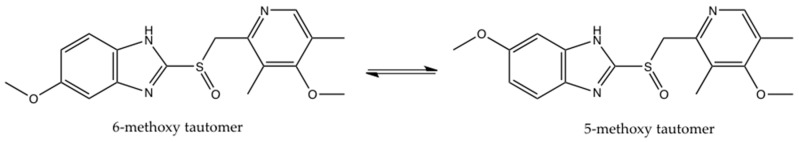
The 5-methoxy and 6-methoxy tautomers of omeprazole.

Omeprazole possesses a single stereogenic center on the sulfur atom and is by definition a racemic mixture of the (*R*) and (*S*) enantiomers. The (*S*)-enantiomer of this API is found in higher concentrations in plasma membrane compared to the (*R*)-enantiomer because it is more slowly and less variably metabolized. The intrinsic clearance is three times lower for esomeprazole, meaning a longer resonance time *in vivo*. The active inhibitor formed is the same for both isomers and as a result they have the same effect on the body [9]. Shown in Figure 1, esomeprazole displays tautomerism of the benzimidazole N-H hydrogen atom, resulting in 5-methoxy and 6-methoxy tautomers. In solution, both tautomers are present, but the 6-methoxy derivative is favoured by nearly 2:1 [10]. According to ^1^H- and ^13^C-NMR studies, the 6-methoxy tautomer is the only form present in the solid state [11]. It has also been confirmed through crystallographic analysis that the 6-methoxy derivative is more stable [7]. This combination of chirality and tautomerism allows for a wide range of possible crystalline structures of this API.

**Figure 2:**
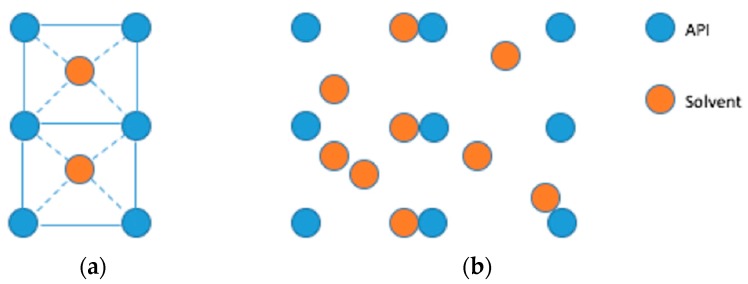
A simplified visualization of (**a**) a “true” solvate *vs.* (**b**) a non-stoichiometric solvate, where the solvent can be one or more types of solvent.

A potential technique to stabilize esomeprazole is to form a solvate by incorporating solvent molecules into the crystal lattice of the API via intermolecular forces. In a “true” solvate, Figure 2a, the solvent molecules become included in the unit cell in stoichiometric amounts. The crystal structure will collapse when solvent is removed from these solvates. Alternatively, solvent molecules can fill the voids and channels of the crystal structure in non-stoichiometric amounts. These types of solvents, shown in Figure 2b, will remain crystalline when solvent is removed [12]. When forming a solvate, one must be vigilant in ensuring that the incorporated solvent is not toxic to the consumer at a given dosage [13]. Minimal research articles have been published exploring the possibility of esomeprazole solvates, though a crystalline form of omeprazole sodium ethanol solvate has been reported [14,15].

Analogous to many prospective APIs, the instability of esomeprazole poses concerns for industry in manufacturing and storage [16]. Since water is present throughout many industrial processes, one attempt at stabilization is through the investigation of possible pseudopolymorphs, such as hydrates. Pharmaceutical hydrates occur when water becomes incorporated into the crystal lattice of an API by means of intermolecular bonding [17]. The small size of water molecule relative to most pharmaceuticals, as well as its hydrogen bonding ability, make it an ideal molecule to incorporate into the crystal lattice [18]. To date, there are patented examples of esomeprazole magnesium in tetrahydrate, trihydrate, dihydrate, monohydrate and hemihydrate forms [19,20]. In a study of esomeprazole magnesium hydrates, it was found that the stability order in water is trihydrate > dihydrate form A > tetrahydrate > dihydrate form B. The solubility order in water was determined to be dihydrate form B > tetrahydrate > dihydrate form A > trihydrate [21].

## 2. Results and Discussion

In the present work, a novel form of esomeprazole magnesium has been characterized by single crystal X-ray diffraction, powder X-ray diffraction (PXRD), ^1^H-NMR spectroscopy, infrared spectroscopy (IR), thermogravimetric analysis (TGA), differential scanning calorimetry (DSC) and dynamic vapor sorption (DVS). The trihydrate form is known to be soluble in methanol, ethanol and 1-butanol [22]. The solubility in ethanol is low, and methanol is toxic to humans, so since 1-butanol is on the FDA-approved list of solvents/excipients, the esomeprazole magnesium water/1-butanol solvate was further investigated. Additionally, crystallization of this solvate was particularly simple and moderately short.

To better understand the bonding and structure of esomeprazole derivatives, various efforts were made at forming single crystals. Plate like single crystals were formed from a solution of amorphous esomeprazole magnesium in 1-butanol in the presence of water at 5 °C after approximately three weeks. The sample was mounted on a Mitegen polyimide micromount with a small amount of Paratone N oil. All X-ray measurements were made on a Bruker Kappa Axis Apex2 diffractometer at a temperature of 110 K. The unit cell dimensions were determined from a symmetry constrained fit of 9907 reflections with 6.4° < 2θ < 46.62°. The data collection strategy was a number of ω and ϕ scans which collected data up to 51.468° (2θ). The frame integration was performed using SAINT [23]. The resulting raw data was scaled and absorption corrected using a multi-scan averaging of symmetry equivalent data using SADABS [24]. The structure was solved by using a dual space methodology using the SHELXT program [25]. All non-hydrogen atoms were obtained from the initial solution. All hydrogen atoms except for water molecules were introduced at idealized positions and were allowed to ride on the parent atom. The hydrogen atoms on the water molecules were added considering hydrogen bonding with nitrogen or oxygen atoms around them. The structural model was fit to the data using full matrix least-squares based on *F*^2^. The calculated structure factors included corrections for anomalous dispersion from the usual tabulation. The structure was refined using the SHELXL-2014 program from the SHELXTL suite of crystallographic software [26]. Graphic plots were produced using the Mercury program suite [27] (a summary of the crystal data is presented in Table 1). CCDC 1468173 contains the supplementary crystallographic data for this paper. These data can be obtained free of charge from The Cambridge Crystallographic Data Centre via www.ccdc.cam.ac.uk/data_request/cif.

**Figure 3:**
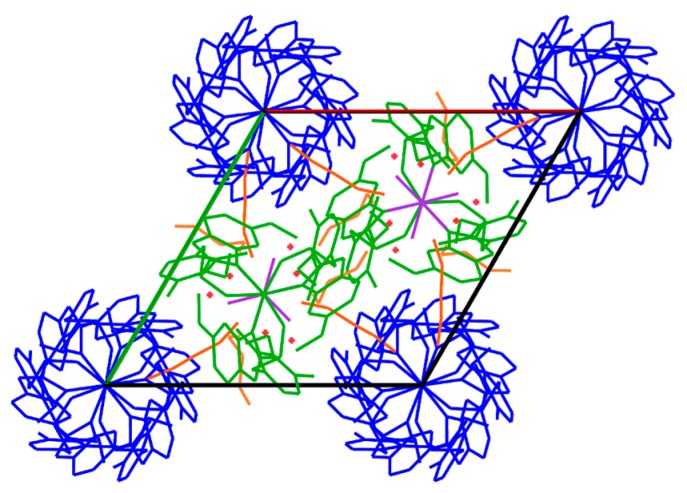
Esomeprazole magnesium molecules crystalize in P6_3_ spaces group alongside with water and 1-butanol molecules; the molecules of the same color are symmetry equivalent.

**Figure 4:**
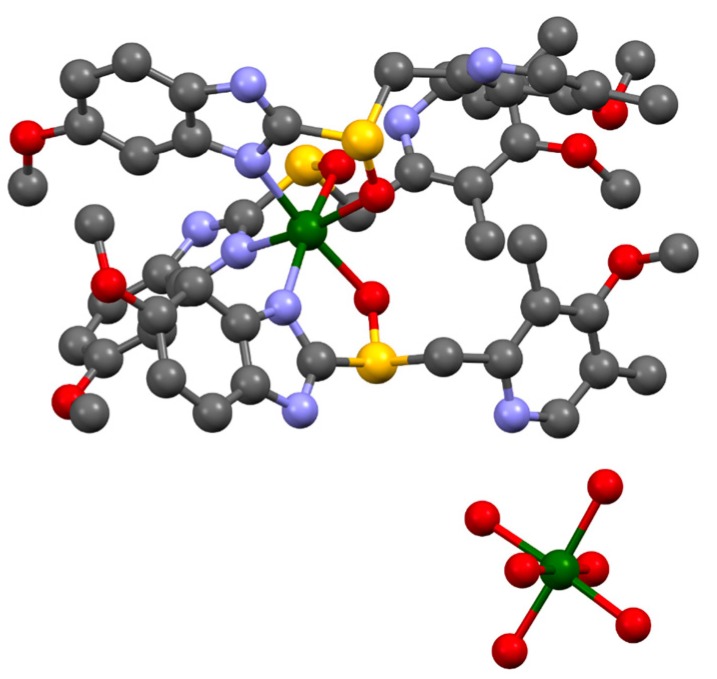
Two types of coordination around the magnesium centers, hydrogen atoms and non-bonded solvents have been omitted for clarity. Mg: green, S: yellow, O: red, N: blue, C: grey.

**Figure 5:**
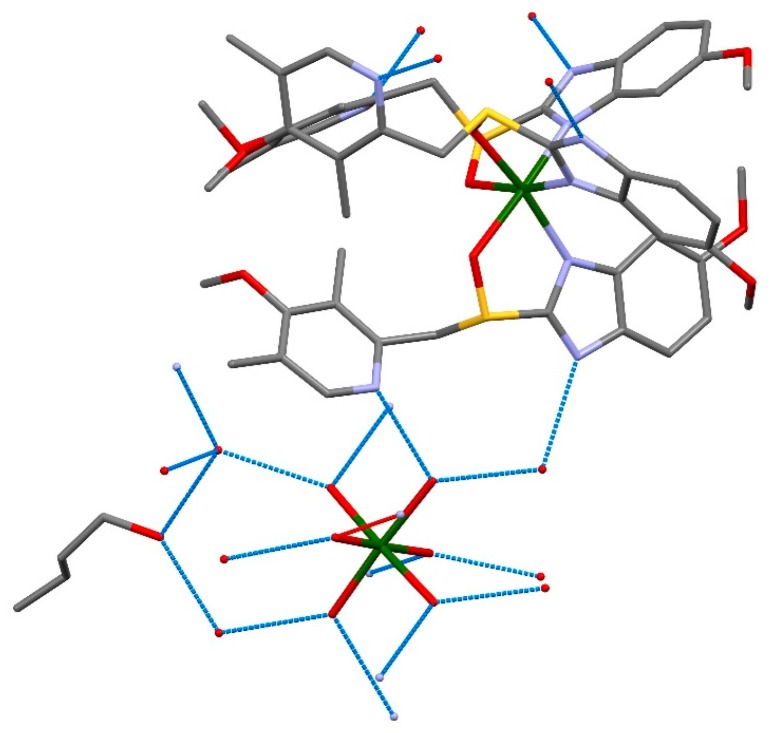
Strong hydrogen bonding between the molecules in the lattice is shown in blue dotted lines; hydrogen atoms have been omitted for more clarity. Mg: green, S: yellow, O: red, N: blue, C: grey.

Molecules of esomeprazole magnesium crystallize in the space group P 6_3_, with *Z* = 2, alongside four water and two 1-butanol molecules (C_126_H_198_Mg_3_N_18_O_36_S_6_) (Figure 3). As shown in Figure 4, coordination around the magnesium centers is octahedral. For every Mg center that has six water molecules around it, there are two magnesium centers each coordinated with three esomeprazole ligands. All of the esomeprazole molecules act as chelating ligands and coordinate the Mg centers with one oxygen as well as one nitrogen atom. This agrees with previous DFT calculations, where omeprazole was shown to favour coordination with Co(II) and Fe(III) through the sulfoxide oxygen and the benzimidazolic nitrogen [28]. All of the six water molecules that are coordinated to the magnesium center show hydrogen bonding either with other water molecules in the lattice or the nitrogen atoms in the esomeprazole ligands (Figure 5). 1-Butanol molecules fill the voids in this lattice and are also hydrogen bonded to the water molecules in the lattice.

**Figure 6:**
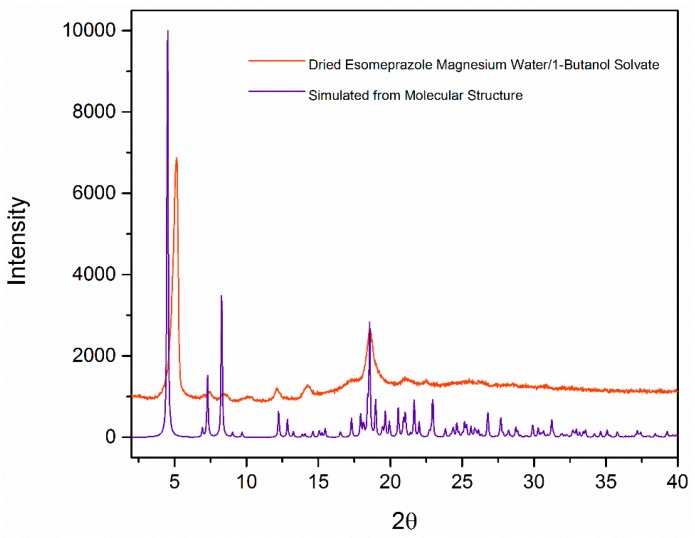
X-ray powder diffraction of esomeprazole magnesium water/butanol solvate calculated from single crystal structure and esomeprazole magnesium water/butanol solvate after drying under vacuum.

From the single crystal structure, the powder pattern was simulated and is shown in Figure 6. This pattern is different than that of the crystals which have been dried under vacuum. In the pattern of the dried sample, the peaks at 2θ = 7° and 8° are much lower in intensity. As well, the simulated peak at 2θ = 4.5° has shifted to 5.1°; however, the peak at 2θ = 18.5° is consistent in both simulated and experimental powder pattern. Some solvent molecules have been removed from the lattice, and as a result, a different polymorph according to the PXRD pattern is formed. This is further proved by other characterization techniques that are discussed below. As seen in the single crystal analysis, water is found in the channels of the lattice along with being bonded to the magnesium cation. The dative covalent bonding of water to the magnesium is much stronger than the hydrogen bonding displayed by the channel water molecules. The change in PXRD pattern shows that the crystal lattice has changed by removing some of the water and 1-butanol molecules. The pattern obtained of the solvate after drying is comparable to that of esomeprazole magnesium dihydrate form A, with equivalent peaks at 2θ = 5° and 18° [29]. The X-ray pattern was consistent for the sample after drying under vacuum at 50 °C for two hours and up to 16 h. The powder diffraction also remained the same for samples that were exposed to air. Since esomeprazole is known to be chemically unstable in air, the ability of the esomeprazole magnesium water/1-butanol solvate to remain crystalline in air highlights a potential advantage for its use in pharmaceutical solid dosage forms.

In order to confirm the occurrence of 1-butanol in the crystal lattice after drying, ^1^H-NMR data was collected from the dry sample in DMSO-*d*_6_ solution. The presence of water and 1-butanol is clear by analysis of the spectrum; esomeprazole: ^1^H-NMR δ 8.25 (m, 1H), 7.37 (d, *J* = 8.6 Hz, 1H), 7.02 (d, *J* = 2.6 Hz, 1H), 6.60 (dd, *J* = 2.5 Hz, 8.4 Hz, 1H), 4.72 and 4.46 [d (AB system, *J* = 12.7 Hz, 2H)], 3.75 (s, 3H), 3.70 (s, 3H) 2.21 (s, 6H); 1-butanol: ^1^H-NMR δ 4.30 (t, 2H), 3.38 (q, 1H), 1.39 (m, 2H) 1.30 (m, 2H) and 0.83 (t, 3H); Water: ^1^H-NMR δ 3.3 (s, 2H), [30,31]. Since it was assumed that all surface water and solvent are removed after sufficient drying, the characteristic peaks were integrated to determine the 1-butanol content. As previously stated, esomeprazole will demonstrate tautomerism in solution. DMSO-*d*_6_, a slow exchange solvent, was utilized in an effort to help reduce these effects in solution [32]. The multiplet occuring at 8.25 ppm is characteristic of esomeprazole, uneffected by tautomerism, and signifies a single proton, while the triplet at 0.84 ppm is representative of the methylene protons of 1-butanol. Integrating these peaks confirmed that there are still 0.3 1-butanol molecules per esomeprazole magnesium in the dried sample. Water, seen at 3.33 ppm, cannot be accurately quantified in this method due to inevitable impurities in the NMR solvent. These impurities arise from DMSO’s affinity for water; it cannot be dried completely over molecular sieves [33].

The structure of esomeprazole magnesium dihydrate/1-butanol solvate contains many different functional groups. IR spectroscopy was utilized to help confirm the presence of esomeprazole, water and 1-butanol after a sample had been dried under vacuum. Table 2 shows the absorption frequencies for these functional groups. The peaks presented are characteristic of esomeprazole with a broad peak at approximately 3000 cm^−1^ from the O-H stretching in water and 1-butanol. The peak representative of the N-H in the benzimidazole, usually seen around 3400 cm^−1^, is not observed. This is due to overlapping with the frequencies of the O-H stretching from coordinated and non-coordinated solvent molecules. Compared to free esomeprazole, no change in frequency is seen for the pyridinic nitrogen. This agrees with the molecular structure determined with single crystal XRD, where esomeprazole is coordinating through benizimidazolic nitrogen and sulfoxide oxygen. The frequencies observed are in close agreement with the calculated IR spectra for omeprazole co-ordinated to different metal centers [28].

**Figure 7:**
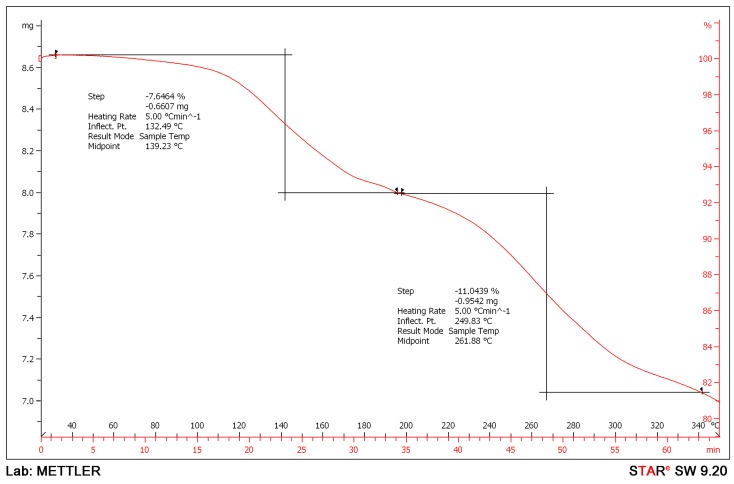
TGA of the title sample showing two mass losses. The first loss of 7.6% is desolvation, while the second loss is decomposition of the esomeprazole molecule.

In an attempt to confirm the total solvent content in the crystal lattice, thermogravimetric analysis (TGA) was performed. The TGA results, shown in Figure 7, revealed two mass losses for this solvate. The first step can be attributed to the loss of solvent in the lattice, while the second step can be accredited to the decomposition of the sample. The desolvation resulted in a loss of 7.6% of the total mass. These results are consistent with the ^1^H-NMR analysis of 0.3 1-butanol and two water molecules per esomeprazole magnesium. The spectrum obtained from TGA confirms that the esomeprazole magnesium water/1-butanol solvate loses solvent from the lattice when dried under vacuum. TGA was also conducted after heating the sample to 120 °C under vacuum, and the same results were acquired. The second mass loss begins at 200 °C, typical for the decomposition of esomeprazole magnesium [29].

**Figure 8:**
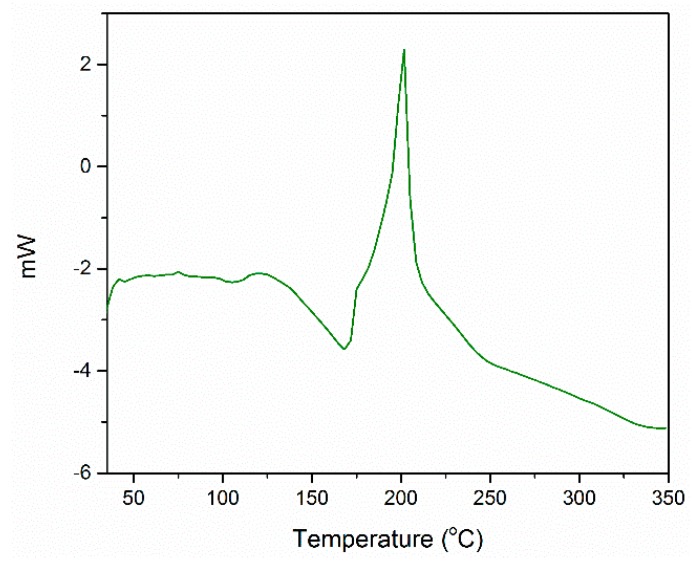
The DSC curve of esomeprazole magnesium water/1-butanol solvate. The curve was obtained from 25 to 350 °C at a heating rate of 5 °C/min. An exotherm characteristic of esomeprazole magnesium is seen at 200 °C as well as an endotherm at 175 °C.

Differential scanning calorimetric analysis was performed on the esomeprazole magnesium water/1-butanol solvate as well (Figure 8). The pattern shown can be described as an exothermic melting via decomposition. Amorphous esomeprazole magnesium is known to display an exothermic peak at approximately 200 °C [36]. When DSC was run on the starting material, the exothermic peak was seen at 181 °C. From Figure 8, the water/1-butanol solvate decomposes at 200 °C. This increase in decomposition temperature implies an increase in stability. The peak shown below is broader than that of the amorphous API; the width at half height is 13.3 °C, while the width is 5.8°C for amorphous esomeprazole magnesium. The broadening agrees with the results from TGA and ^1^H-NMR that show solvent is still present in the lattice. In addition to this peak, the DSC curve of the title compound also shows an endotherm at 175 °C. This curve matches known results for esomeprazole magnesium dihydrate, which aids in validating the assumption that some of the solvent is being removed after vacuum drying, leaving esomeprazole magnesium dihydrate with some 1-butanol molecules [19].

**Figure 9:**
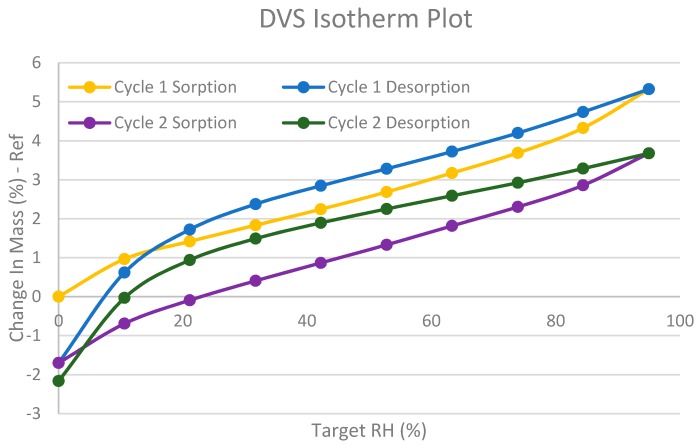
Esomeprazole magnesium water/1-butanol adsorption of water at increasing humidity followed by desorption at decreasing humidity. The cycle is repeated to give two isotherms.

The stability of this compound is further investigated by DVS; the subsequent isotherm is shown in Figure 9. In sorption cycles 1 and 2, there is a 5.3% mass increase. The adsorption/desorption of water is a reversible process in the title compound. Since the sample reverted to the initial mass, the water adsorbed can be said to be on the surface of the API. The behaviour of this isotherm implies that the structure of the title compound is highly crystalline.

## 3. Materials and Methods

Esomeprazole magnesium (MW = 713.12 g/mol) was provided by Apotex Pharmachem Inc. (Brantford, ON, Canada) and kept in an inert environment when in storage. 1-Butanol was purchased from Fischer Scientific (Waltham, MA, USA) and used in crystallization. All ^1^H-NMR spectra were obtained from solutions in DMSO-*d*_6_ purchased from Sigma Aldrich (Oakville, ON, Canada).

An Apex II Diffractometer (Bruker, East Milton, ON, Canada) with Mo X-ray radiation was used to obtain the crystal structure of the title compound. Powder X-ray Diffraction was obtained from a Miniflex instrument (Rigaku, Auburn Hills, MI, USA) with Cu X-ray radiation. Scans were run at a speed of 1.00°/min for 2θ values of 2.00°–40.00°. Thermogravimetric analysis and differential scanning calorimetry were conducted on the STAR^e^ 851^e^ and 822^e^ instruments (Mettler-Toledo, Mississauga, ON, Canada), respectively, at a heating rate of 5 °C/min from 25 to 350 °C. ^1^H-NMR spectra were collected on a Mercury VX 400 MHz machine (Varian Inc., Santa Clara, CA, USA). IR results were obtained over 32 scans from 4000 cm^−1^ to 500 cm^−1^ using a Nicolet 6700 FT-IR spectrometer. DVS-1000, from Surface Measurement Systems Ltd. (Allentown, PA, Canada), was used to obtain results for dynamic vapor sorption.

The esomeprazole magnesium water/butanol solvate was obtained by dissolving 0.750 g of amorphous esomeprazole magnesium in 7.0 mL of 1-butanol. Once in solution, it was filtered to obtain a clear solution, then 1.0 mL of distilled water was added and stirred until miscible. The solution was kept overnight at 5.0 °C. Crystalline powder was formed which was filtered and washed with 1-butanol. Surface solvent was removed by heating under vacuum at 50–55 °C up to 16 h. The single crystal sample was obtained by dissolving 0.700 g of amorphous esomeprazole magnesium in 40.0 mL of 1-butanol. To the clear solution, 2.0 mL of distilled water was added. The solution was then left at 5.0 °C for 2–3 weeks until single crystals had formed.

## 4. Conclusions

This is the first reported single crystal structure that displays esomeprazole coordinated to a metal center. Analysis of the crystal structure shows that water is bound to magnesium cation as well filling the channels of the lattice. The covalently bonded waters are much more strongly retained in the lattice than those maintained by hydrogen bonding. The discovery of this esomeprazole magnesium solvate could have applicability to the development of improved pharmaceutical solid dosage forms.

## Figures and Tables

**Table 1:** Summary of Crystal Data.

Formula	C_126_H_198_Mg_3_N_18_O_36_S_6_
Formula Weight (g/mol)	2806.30
Crystal Dimensions (mm)	0.185 × 0.142 × 0.092
Crystal System	Hexagonal
Space Group	P 6_3_
Temperature, K	110
*a*, Å	14.701(4)
*b*, Å	14.701
*c*, Å	39.123(11)
α, °	90
β, °	90
γ, °	120
V, Å^3^	7323(5)
Z	2
F(000)	3000
ρ (g/cm)	1.273
λ, Å, (MoKα)	0.71073
μ, (cm^−1^)	0.185
R_1_	0.0674
wR_2_	0.1733
GOF	1.032
Maximum shift/error	0.000
Min & Max peak heights on final ΔF Map (*e^−^*/Å)	−0.497, 0.487

**Table 2:** IR spectroscopy absorption frequencies and corresponding functional groups.

Functional Group	Absorption Frequency (cm^−1^)	Type of Vibration
sulfoxide	1076	S=O stretching [34]
methoxy	1199	C-O stretching [34]
methoxy	1228	C-O stretching [34]
amine	1409	N-H bending [28]
pyridine	1569	C=N stretching [28]
benzimidazole	1612, 1588	C=N stretching [28]
aromatic	2970	C=C-H asymmetric stretching [34]
alcohol/water	3100–2800	O-H stretching [35]

## Abbreviations

API	Active pharmaceutical ingredient
FDA	US Food and Drug Administration
DFT	Density functional theory
DSC	Differential scanning calorimetry
DVS	Dynamic vapor sorption
IR	Infrared
NMR	Nuclear magnetic resonance
PXRD	Powder X-ray diffraction
TGA	Thermogravimetric analysis
